# Frequency and spectrum of *MT-TT* variants associated with Leber’s hereditary optic neuropathy in a Chinese cohort of subjects

**DOI:** 10.1080/23802359.2019.1627921

**Published:** 2019-07-12

**Authors:** Yuanyuan Lyu, Man Xu, Jie Chen, YanChun Ji, Min-Xin Guan, Juanjuan Zhang

**Affiliations:** a School of Ophthalmology and Optometry, Wenzhou Medical University, Wenzhou, Zhejiang, China;; b School of Laboratory Medicine and Life Sciences, Attardi Institute of Mitochondrial Biomedicine, Wenzhou Medical University, Wenzhou, Zhejiang, China;; c School of Medicine, Institute of Genetics, Zhejiang University, Hangzhou, Zhejiang, China

**Keywords:** Leber’s hereditary optic neuropathy (LHON), MT-TT gene, variant, spectrum, Chinese

## Abstract

Leber’s hereditary optic neuropathy (LHON) is a maternally inherited eye disease. In our previous investigations, we have reported the spectrum and frequency of mitochondrial *MT-ND1*, *MT-ND4* and *MT-ND6* gene in Chinese LHON population. This study aimed to assess the molecular epidemiology of *MT-TT* mutations in Chinese families with LHON. A cohort of 352 Chinese Han probands lacking the known LHON-associated mtDNA mutations and 376 control subjects underwent molecular analysis of mtDNA. All variants were evaluated for evolutionary conservation, structural and functional consequences. Fifteen variants were identified in the *MT-TT* gene by mitochondrial genome analysis of LHON pedigrees, which was substantially higher than that of individuals from general Chinese populations. The incidences of the two known LHON-associated mutations, m.15927G > A and m.15951A > G, were 2.27% and 1.14%, respectively. Nine putative LHON-associated variants were identified in 20 probands, translated into 2.1% cases of this cohort. Moreover, mtDNAs in 41 probands carrying the *MT-TT* mutation(s) were widely dispersed among nine Eastern Asian haplogroups. Our results suggest that the *MT-TT* gene is a mutational hotspot for these 352 Chinese families lacking the known LHON-associated mutations. These data further showed the molecular epidemiology of *MT-TT* mutations in Chinese Han LHON pedigrees.

## Introduction

Leber’s hereditary optic neuropathy (LHON, #535000) is a maternally inherited mitochondrial disorder leading to vision failure by the preferential loss of retinal ganglion cells (RGCs), and marked adult male bias (Yu-Wai-Man et al. 2011; Jurkute and Yu-Wai-Man 2017). The minimum prevalence of visual impairment due to LHON was 3.22 per 100,000 in northeast of England (Yu-Wai-Man et al. 2003). Maternal inheritance of LHON indicated the involvement of mutations in mitochondrial DNA (mtDNA) (Wallace et al. 1988; Howell 2003). The human mitochondrial genome is a 16,569 bp, double-stranded, circular molecule that codes for 13 subunits of respiratory chain complexes, two rRNAs (12S and 16S rRNA) and 22 mitochondrial tRNAs (Andrews et al. 1999). The majority of subjects with LHON (90%–95%) harbor one of three primary LHON-associated mtDNA mutations (m.3460G > A, m.11778G > A, and m.14484T > C) in some western countries (Brown et al. 1995; Mackey et al. 1996; Mashima et al. 1998), while these mutations are only responsible for 38.3% and 41.1% cases in two large cohorts of Chinese LHON subjects, respectively (Jia et al. 2006; Liang et al. 2014; Jiang et al. 2015; Ji et al. 2016). Thus, other mtDNA genes including mitochondrial tRNA genes are the hotspots associated with LHON (Ruiz-Pesini et al. 2007; Zheng et al. 2012; Xue et al. 2016). In our previous four investigations, four tRNA mutations (*MT-TM* 4435A > G, *MT-TE* 14693A > G, *MT-TT* 15927G > A and 15951A > G) have been identified as LHON-associated mutations (Li et al. 2006; Qu et al. 2006; Tong et al. 2007; Zhang et al. 2018). These studies tested only in relatively small sized samples of pedigrees, while the association of *MT-TT* mutations with LHON in a large population remains to be explored. The purpose of this present study was to perform a comprehensive test of the hypothesis that *MT-TT* variants play an important role in the pathogenesis of LHON. For this objective, we recruited a cohort of 352 genetically unrelated Chinese Han patients with LHON (269 males and 82 females) and 376 Chinese control subjects performed the Sanger sequence analysis of the DNA fragments spanning *MT-TT* gene, and then, investigated mutational spectrum and incidences of *MT-TT* gene. This analysis showed the identification of 15 nucleotide changes among *MT-TT* gene. To identify deleterious mutations from polymorphisms, these variants were further evaluated using the criteria shown in the previous studies (Bandelt et al. 2009; Zheng et al. 2012; Kirchner and Ignatova 2015; Xue et al. 2016). These analyses showed that 9 tRNA variants might have higher evolutionary conservation index, structural and functional alterations. Moreover, these mtDNAs of 20 subjects carrying the putative variants were assigned to the Asian mtDNA haplogroups using the nomenclature of mtDNA haplogroups (Tanaka et al. 2004; Kong et al. 2006).

## Materials and methods

### Subjects

A totally of 352 unrelated Han Chinese LHON subjects lacking the known LHON-associated mtDNA mutations were recruited for this investigation. This study was in compliance with the Declaration of Helsinki (Liang et al. 2014; Jiang et al. 2015; Ji et al. 2016). The institutional review boards of Wenzhou Medical University and Zhejiang University approved this study. A cohort of 376 Chinese control subjects obtained from the same areas were screened for the presence of mtDNA variants.

### Ophthalmologic examinations

These probands and other members of these families received ophthalmological examinations at School of Ophthalmology and Optometry, Eye Hospital, Wenzhou Medical University and were diagnosed as LHON. The degree of visual impairment was defined according to the visual acuity as follows: normal > 0.3, mild = 0.3–0.1, moderate <0.1–0.05, severe <0.05–0.02, and profound <0.02 (Qu et al. 2009; Liu et al. 2011).

### Mutational analysis of mitochondrial genomes

Genomic DNA was isolated from whole blood of participants (352 probands lacking the known LHON-associated mutations and 376 Chinese control subjects) using QIAamp DNA Mini Kit (Qiagen). Subjects’ DNA fragments spanning the *MT-TT* gene were amplified, purified and subsequently analyzed by direct sequencing in an ABI 3700 automated DNA sequencer using the BigDye Terminator Cycle sequencing reaction kit (Applied Biosystems) (Jia et al. 2018; Zhang et al. 2018). These sequence results were compared with the revised Cambridge Reference Sequence (rCRS, NC_012920.1) (Bandelt et al. 2014). For defining the mitochondrial haplogroups, the entire mitochondrial genomes of 41 subjects with *MT-TT* mutations were PCR amplified in 24 overlapping fragments using sets of the light (L) strand and the heavy (H) strand oligonucleotide primers, as described previously (Rieder et al. 1998). The analysis of variants was evaluated according to the previous description (Zou et al. 2010; Zhang et al. 2011, 2012).

### Evolutionary conservation and structural analysis

Evolutionary conservation analysis for certain mtDNA variant was performed by comparing human mtDNA to 43 different vertebrate species, as shown in our previous studies (Ruiz-Pesini and Wallace 2006; Carelli et al. 2017). The conservation index (CI) of certain variant was defined by the percentage of species for a list of 44 different vertebrate species (including *Homo* species). The secondary cloverleaf and tertiary structure of human *MT-TT* were analyzed by the online software (Sprinzl and Vassilenko 2005; Jühling et al. 2009; Lott et al. 2013).

### Haplogroup classification

The mtDNA sequences of eight probands carrying m.15927G > A, four subjects carrying the m.15951A > G mutation, as well as 20 subjects carrying the putative *MT-TT* variants are assigned to the Asian mtDNA haplogroups by using the nomenclature of mitochondrial haplogroups (Kong et al. 2006; Zou et al. 2010).

### Statistical analysis

Statistical analysis was performed by the *χ*^2^ test contained in Microsoft Office Excel (Version 2017). *p* value indicates the significance, according to the *χ*^2^ test, of the difference between mutant and control mean. Differences were considered significant at a *p* < .05.

## Results

### Study samples

**Figure 1.**
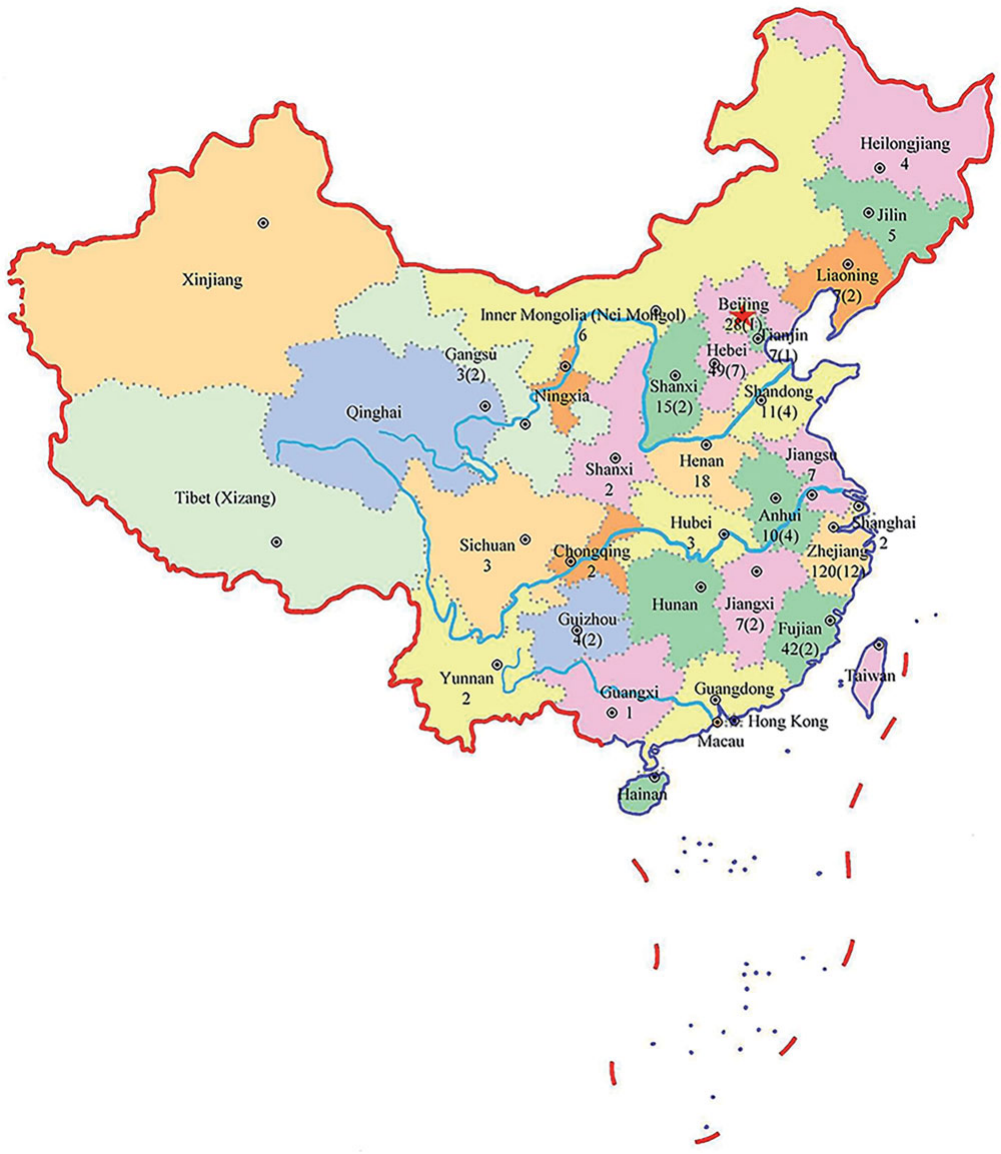
Geographic locations of 352 Han Chinese subjects with LHON. The numbers in parenthesis indicate 41 patients with *MT-TT* mutations.

The study samples lacking the known LHON-associated mtDNA mutations consisted of 269 males and 83 females. All participants were Han Chinese subjects recruited from eye clinics of 25 provinces in China, as shown in Figure 1. Ophthalmologic evaluation showed that all affected subjects exhibited the variable severity and age at onset of optic neuropathy. Of these, 38 subjects exhibited profound visual impairment, 50 subjects had severe visual impairment, 53 individuals suffered from moderate visual impairment, and 212 subjects had mild visual impairment. The age at onset of optic neuropathy ranged from 1 to 52 years, with an average of 17.5 years. Comprehensive family medical histories of those probands showed no other clinical abnormalities, including diabetes, muscular diseases, hearing loss, and other neurological disorders.

### Mutational analysis of MT-TT gene

Deoxyribonucleic acid fragments spanning *MT-TT* gene were PCR-amplified from genomic DNA of 352 Chinese subjects with LHON and 376 Han Chinese control individuals. Each fragment was purified and subsequently analyzed by DNA sequencing. Comparison of the resultant sequences in 352 affected subjects with the Cambridge consensus sequence identified 15 known nucleotide changes in the *MT-TT* gene (Lott et al. 2013), but only seven variants in this gene were identify in 376 control individuals, as shown in the Table 1. The m.15927G > A and m.15951A > G mutations were the two known LHON-associated *MT-TT* gene mutations (Li et al. 2006; Zhang et al. 2018). All the nucleotide changes were identified by sequence analysis of both strands and appeared to be homoplasmy. In the mutational screening, no *MT-TT* nucleotide changes were detected in the 311 patients and 353 controls, while at least one *MT-TT* variant was identified in 41 affected subjects and 23 control individuals (*p* = .0085). That indicates that *MT-TT* gene is a mutational hotspot for Chinese LHON pedigrees. Among these, 12 individuals carried one of the known LHON-associated *MT-TT* mutations, including eight subjects carrying the m.15927G > A mutation and four individuals with the m.15951A > G mutation. Furthermore, the nine variants carrying in twenty probands were considered as putative LHON-associated variants and other four variants belonged to the polymorphisms.

**Table 1.** Variants in the *MT-TT* gene in 352 Chinese subjects with LHON.

Position	Replacement	Location	Site^a^	WC base-pairs	Conservation index (%)^b^	No. of affected subjects (no./352)	No. of controls (no./376)	Haplogroup specific variant^c^	Reported (population context)^d^	Reported (disorder context)^d^	Reported (Mitomap)^e^
Known LHON-associated mutations											
15927	G-A	the anticodon stem	42	↓C-G	75.45	8(2.27)	0	Yes (B5b, G3b, etc.)	Yes	Yes	Yes
15951	A-G	the acceptor stem	71	↓U-A	70.45	4(1.14)	0	Yes (D4b1)	Yes	Yes	Yes
Putative LHON-associated variants											
15900	T-C	the DHU loop	13		72.73	1(0.28)	1(0.27)	Yes (K1c1b)	Yes	No	Yes
15901	A-G	the DHU loop	14		100.00	1(0.28)	0	Yes (D4t)	No	No	Yes
15908	T-C	DHU-stem	23	↓U-A	93.18	1(0.28)	1(0.27)	Yes (M33a, F4a2)	Yes	Yes	Yes
15924	A-G	the anticodon stem	39	↓U-A	90.91	9(2.56)	3(0.71)	Yes (D4e1a, M13, etc.)	Yes	Yes	Yes
15928	G-A	the anticodon stem	43	↓C-G	77.27	3(0.85)	2(0.53)	Yes (C7b, Z3a, etc.)	Yes	Yes	Yes
15931	A-C	the variable loop	46		97.73	1(0.28)	0	No	No	No	No
15940	T-Del	T-loop	56		22.73	1(0.28)	0	Yes (G4)	Yes	Yes	Yes
15943	T-C	T-stem	63	↓U-A	79.55	2(0.57)	1(0.27)	No	No	Yes	Yes
15949	G-A	the acceptor stem	69	↓C-G	88.64	1(0.28)	0	No	No	No	Yes
Other variants											
15907	A-G	the DHU loop	22		65.91	1(0.28)	0	Yes (U2e)	Yes	No	Yes
15930	G-A	the variable loop	45		25.00	2(0.57)	13(3.46)	Yes (B4d, C6, etc.)	Yes	Yes	Yes
15938	C-T	T-loop	54		40.91	1(0.28)	0	Yes (M39)	Yes	No	Yes
15941	T-C	T-loop	61		47.73	5(1.42)	2(0.53)	Yes (B4c1c)	Yes	No	Yes

^a^ Numbers represent the nucleotide positions according to the tRNAdb numbering system (Sprinzl and Vassilenko 2005) and mitotRNAdb http://mttrna.bioinf.uni-leipzig.de/mtDataOutput (Juhling et al. 2009).

^b^ Conservation index indicates the conservative properties of the nucleotides in 44 species.

^c^ The column ‘‘Haplogroup specific variant” refers to the presence or absence of the corresponding variants in the world mtDNA phylogeny displayed at http://www.phylotree.org/tree/index.htm (mtDNA tree Build 17; 18 Feb 2016).

^d^ The search was performed on 18 April 2019 following the described strategy (Bandelt et al. 2009).

^e^ According to MITOMAP (http://www.mitomap.org/MITOMAP). Database of reported mitochondrial DNA base substitution diseases: rRNA/tRNA mutations was last edited on March 06, 2019.

### Evaluation of the MT-TT variants

**Figure 2.**
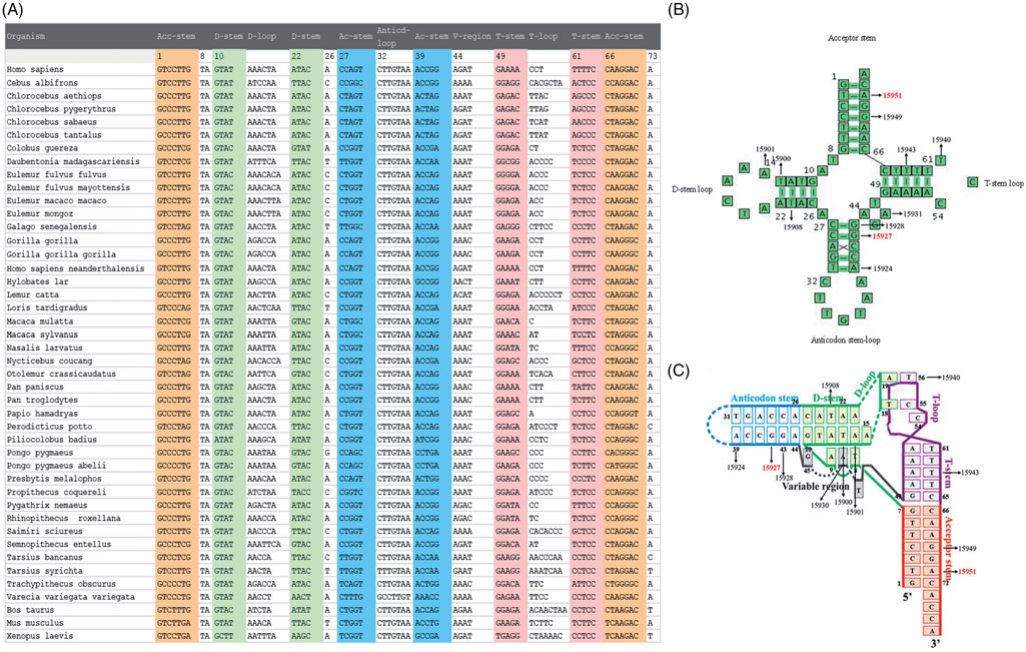
Analysis of *MT-TT* variants. (A) Sequence alignment of 44 vertebrates *MT-TT*; summary of two LHON-associated mutations (Red) and nine putative LHON-associated variants at the cloverleaf (B) and tertiary (C) structures of canonical tRNA^Thr^.

These variants in *MT-TT* were first evaluated by the phylogenetic analysis of these variants and amino acid sequences from other 43 vertebrates. The conservation index among these residues ranged from 22.7% to 100%, as shown in Table 1. Of these, conservation indexes of 9 variants were greater than 70%, with potential functional significance (Figure 2) (Ruiz-Pesini and Wallace 2006). As shown in Table 1, eight variants were absent in 376 Chinese controls, while the frequencies of 7 variants ranged from 0.27% to 3.46% in this control population. Furthermore, we analyzed the structural alteration of tRNA^Thr^ by these variants based on the predicated secondary and tertiary structure. As shown in Figure 2, cloverleaf and tertiary structure of human *MT-TT* consists of the acceptor stem, DHU-stem, D-loop, the anticodon stem, anticodon loop, variable region, T-stem and T-loop. Two variants (m.15927G > A and m.15949G > A), localized at the acceptor stem, and three variants (m.15927G > A, m.15924A > G, and m.15928G > A) resided at the anticodon stem. In addition to the known LHON-associated m.15927G > A and m.15951A > G mutations, six variants (m.15908T > C, m.15924A > G, m.15928G > A, m.15940DelT, m.15943T > C, m.15949G > A), which were absent in 376 Chinese controls and whose conservation indexes were greater than 70%, were the putative LHON-associated variants. On the other hand, four other variants (m.15907A > G, m.15930G > A, m.15938C > T, m.15941T > C), which were present in the controls or lower conservation indexes, appeared to be the polymorphisms.

### Characterization of 20 Chinese probands

**Figure 3.**
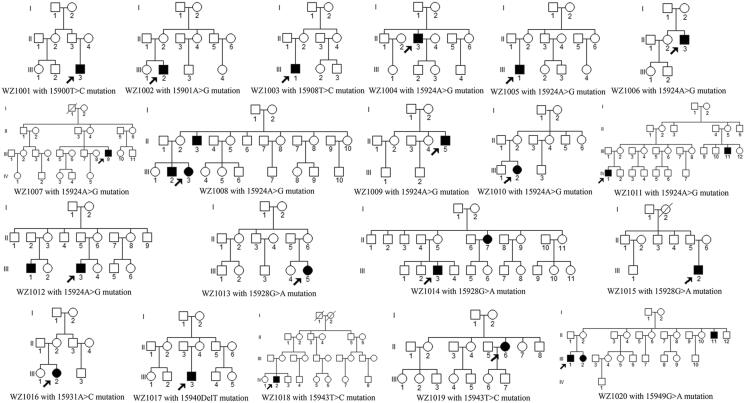
Twenty Han Chinese pedigrees with LHON. Visually-impaired individuals indicated by filled symbols. Arrowhead denotes probands.

**Figure 4.**
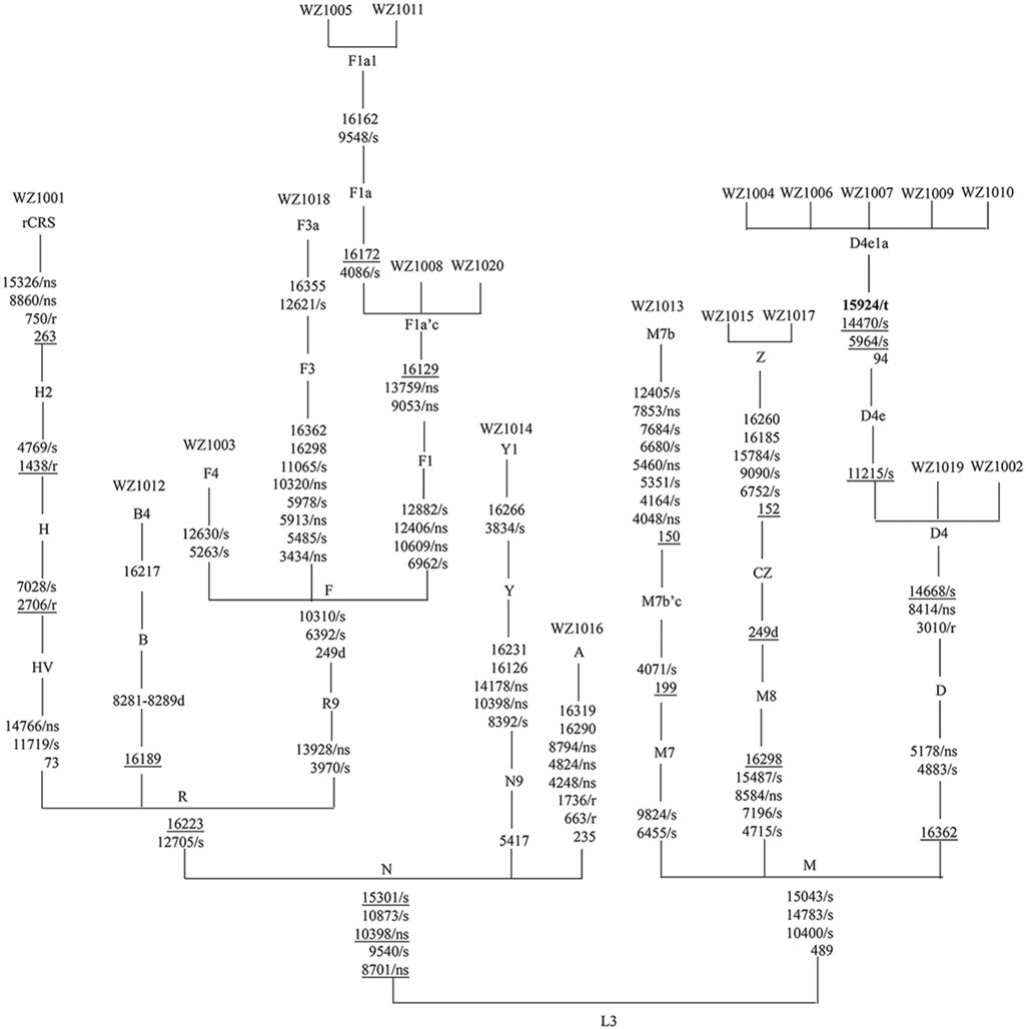
Classification tree of 20 complete mtDNA sequences, plus the revised Cambridge reference sequence (rCRS). The synonymous and non-synonymous coding-region variants in the mtDNA sequences are denoted by “/s” and “/ns,” respectively. Variants in the ribosomal RNA genes and tRNA genes are denoted by “/r” and “/t.” Recurrent mutations are underlined.

Comprehensive medical histories of 20 probands carrying one of nine putative LHON-associated *MT-TT* variants and other members in these families showed no other clinical abnormalities, including diabetes, muscular diseases, hearing loss, and neurological disorders. As shown in Table 2 and Figure 3, these families exhibited a wide range of severity, age at onset, and penetrance of optic neuropathy. Of these, only one matrilineal relative per family in fifteen pedigrees suffered from optic neuropathy, while five pedigrees (WZ1008, WZ1011, WZ1012, WZ1014, and WZ1020) had a history of optic neuropathy. The putative variants were first examined in all available members of these pedigrees. The mtDNA mutations were presented in matrilineal relatives in each family in the homoplasmic form, but not in other members of every family. To assess the contribution that mtDNA variants make toward the variable penetrance and expressivity of optic neuropathy in these Chinese pedigrees, we analyzed entire mtDNA sequences in 20 Chinese probands (Genbank accession numbers: MK795825-MK795844). These affected individuals exhibited distinct sets of mtDNA polymorphisms including 217 known and 4 novel variants (Table 3), belonging to Eastern Asian haplogroups A, D4, F, H2, G2, M7, B2, Y1 and Z, respectively (Figure 4) (Kong et al. 2006). These variants in RNAs and polypeptides were further evaluated by phylogenetic analysis of these variants and sequences from other 43 vertebrates. The *MT-ND1* 3391G > A (G29S), *MT-ND4* 11204T > C (F149L), and *MT-ND6* 14178T > C (I166V) variants showed high evolutionary conservation in these species. There were no other known LHON-associated mutations found.

**Table 2.** Summary of the clinical and molecular data for 20 Han Chinese probands carrying one of the putative *MT-TT* variants.

Putative variants	Proband	Sex	Age at onset, yr	Visual acuity, right/left	Level of visual loss	Family of visual loss	mtDNA haplogroup
m.15900T > C	WZ1001-III-3	M	8	0.1/0.2	Mild	No	H2
m.15901A > G	WZ1002-III-2	M	20	0.3/0.1	Mild	No	D4
m.15908T > C	WZ1003-III-1	M	15	0.2/0.3	Mild	No	F4
m.15924A > G	WZ1004-II-3	M	1	0.15/0.1	Mild	No	D4e1a
	WZ1005-III-1	M	13	0.02/0.01	Profound	No	F1a1
	WZ1006-II-3	M	21	0.01/0.01	Profound	No	D4e1a
	WZ1007-III-9	M	19	0.19/0.1	Mild	No	D4e1a
	WZ1008-III-3	F	25	0.01/0.02	Profound	Yes	F1a′c
	WZ1009-II-5	M	17	0.3/0.3	Mild	No	D4e1a
	WZ1010-III-2	F	3	0.1/0.1	Mild	No	D4e1a
	WZ1011-IV-1	M	20	0.02/0.04	Serve	Yes	F1a1
	WZ1012-III-3	M	5	0.011/0.04	Serve	Yes	B4
m.15928G > A	WZ1013-III-5	F	15	0.1/0.2	Mild	No	M7b
	WZ1014-III-3	M	41	0.2/0.3	Mild	Yes	Y1
	WZ1015-III-2	M	12	0.1/0.04	Mild	No	Z
m.15931A > C	WZ1016-III-2	F	18	0.1/0.3	Mild	No	A
m.15940DelT	WZ1017-III-3	M	20	0.2/0.2	Mild	No	Z
m.15943T > C	WZ1018-IV-2	M	18	0.02/0.03	Serve	No	F3a
	WZ1019-II-6	F	35	0.1/0.1	Mild	No	D4
m.15949G > A	WZ1020-III-1	M	23	0.01/0.01	Profound	Yes	F1a′c

**Table 3.** mtDNA variants in 20 Han Chinese probands carrying one of the putative *MT-TT* variants.

Gene	Position	Replacement	Number ofcontrol(Number/376)	WZ1001	WZ1002	WZ1003	WZ1004	WZ1005	WZ1006	WZ1007	WZ1008	WZ1009	WZ1010	WZ1011	WZ1012	WZ1013	WZ1014	WZ1015	WZ1016	WZ1017	WZ1018	WZ1019	WZ1020	Previouslyreported^a^
D-loop	73	A-G	302	G	G	G	G	G	G	G	G	G	G	G	G	G	G	G	G	G	G	G	G	Yes
	94	G-A	2				A		A	A		A	A											Yes
	146	T-C	37			C											C							Yes
	150	C-T	63													T								Yes
	151	C-T	8																	T				Yes
	152	T-C	75									C						C		C				Yes
	153	A-G	6																	G				Yes
	189	A-G	1				G																	Yes
	194	C-T	8				T																	Yes
	195	T-C	18	C																				Yes
	199	T-C	23													C								Yes
	200	A-G	5																			G		Yes
	204	T-C	23								C	C									C		C	Yes
	207	G-A	28									A									A			Yes
	235	A-G	25																G					Yes
	249	DelA	69			Del A		Del A			Del A			Del A				Del A		Del A	Del A		Del A	Yes
	263	A-G	303	G		G	G	G	G	G	G	G	G	G	G	G	G	G		G	G	G	G	Yes
	281	A-G	1			G																		Yes
	310	T-TC/CTC	247	TC	TC	CTC	CTC	TC	TC	CTC	CTC	CTC	CTC	TC	TC	CTC	CTC	CTC	TC	TC	CTC	TC	CTC	Yes
	489	T-C	116		C		C		C	C		C	C			C		C				C		Yes
	508	A-G	0									G												Yes
	523	Del C	101			Del C		Del C			Del C			Del C	Del C				Del C					Yes
	524	Del A	91			Del A		Del A			Del A			Del A	Del A				Del A					Yes
	16025	T-C	0																				A	Yes
	16092	T-C	21						C	C		C	C											Yes
	16093	T-C	23			C															C			Yes
	16126	T-C	7														C							Yes
	16129	G-A	72					A			A		A	A	A	A							A	Yes
	16162	A-G	12					G						G										Yes
	16172	T-C	34					C			C			C									C	Yes
	16176	C-T	1	T			T										T							Yes
	16182	A-C	38												C									Yes
	16183	A-C	87												C									Yes
	16185	C-T	10			T												T		T				Yes
	16189	T-C	112												C									Yes
	16192	C-T	13													T								Yes
	16207	A-G	2			G																		Yes
	16217	T-C	22												C									Yes
	16223	C-T	183	T	T		T		T	T		T	T			T		T	T	T		T		Yes
	16231	T-C	3														C							Yes
	16232	C-A	2	A																				Yes
	16256	C-T	7																			T		Yes
	16260	C-T	18															T		T	T			Yes
	16266	C-T	2														T							Yes
	16274	G-A	13																					Yes
	16288	T-C	0													C								Yes
	16290	C-T	23																T					Yes
	16298	T-C	43															C		C	C			Yes
	16302	A-G	0																	G				Yes
	16304	T-C	47			C		C			C			C									C	Yes
	16311	T-C	56																			C		Yes
	16319	G-A	44																A					Yes
	16355	C-T	6	T																	T			Yes
	16362	T-C	114		C		C		C	C		C	C						C		C	C		Yes
	16399	A-G	6			G		G						G			G			G				Yes
	16519	T-C	146	C		C		C			C			C			C					C		Yes
12S rRNA	663	A-G	20																G					Yes
	709	G-A	67																		A			Yes
	750	A-G	340	G	G	G	G	G	G	G	G	G	G	G	G	G	G	G	G	G	G	G	G	Yes
	794	T-C	0																	C				Yes
	813	A-G	0	G																				Yes
	1438	A-G	339	G	G	G	G	G	G	G	G	G	G	G	G	G	G	G	G	G	G	G	G	Yes
16S rRNA	1736	A-G	19																G					Yes
	1864	A-G	0									G	G											Yes
	2706	A-G	343	G	G	G	G	G	G	G	G	G	G	G	G	G	G	G	G	G	G	G	G	Yes
	3010	G-A	60		A		A		A	A		A	A									A		Yes
	3290	T-C	2			C																		Yes
ND1	3316	G-A (Ala4Thr)	9				A		A	A		A	A											Yes
	3368	T-C	0								C												C	Yes
	3372	C-T	0										T											Yes
	3391	G-A (Gly29Ser)	1						A															Yes
	3434	5																		G			Yes	
	3480	G-A	0										A											Yes
	3834	G-A	3														A							Yes
	3970	C-T	58			T		T			T			T							T			Yes
	4048	G-A (Asp248Asn)	13													A								Yes
	4071	C-T	24													T								Yes
	4086	C-T	16					T						T										Yes
	4163	0								C													No	
	4164	A-G	3													G								Yes
	4248	T-C(Ile314Ile)	20																C					Yes
ND2	4685	A-G	0																				G	Yes
	4702	A-G	0																				C	Yes
	4715	A-G	27								G							G		G			G	Yes
	4722	A-G	0																				G	Yes
	4769	A-G	343	G	G	G	G	G	G	G	G	G	G	G	G	G	G	G	G	G	G	G	G	Yes
	4824	A-G(Thr119Ala)	21																G					Yes
	4870	0																				G	No	
	4883	C-T	77		T		T		T	T		T	T									T		Yes
	5093	T-C	1												C									Yes
	5139	A-G	0																				G	Yes
	5178	C-A (Leu237Met)	77		A		A		A	A		A	A									A		Yes
	5263	C-T	3			T																		Yes
	5304	C-T	0																				T	Yes
	5315	A-G	0					G						G										Yes
	5351	A-G	10													G								Yes
	5417	G-A	29														A							Yes
	5460	G-A (Ala331Thr)	11													A								Yes
NC3	5585	G-A	9																		A			Yes
NC5	5894	A-G	5																		G			Yes
CO1	5913	5																		A			Yes	
	5964	T-C	1				C		C	C		C	C											Yes
	5978	A-G	9																		G			Yes
	6272	A-G	0														G							Yes
	6392	T-C	56			C		C			C			C							C		C	Yes
	6455	C-T	0													T								Yes
	6515	A-G	3								C												C	Yes
	6674	T-C	0	C																				Yes
	6680	T-C	9													C								Yes
	6734	G-A	29						A															Yes
	6752	A-G	10															G		G				Yes
	6962	G-A	29					A			A			A									A	Yes
	7028	C-T	338	T	T	T	T	T	T	T	T	T	T	T	T	T	T	T	T	T	T	T	T	Yes
	7196	C-A	34															A		A				Yes
	7364	A-G	4												G									Yes
	7424	A-G	1														G							Yes
CO2	7684	T-C	12													C								Yes
	7775	G-A	0															A						Yes
	7853	G-A (Val90Ile)	17													A								Yes
	7975	A-G	0	G																				Yes
	8119	T-C	1							C														Yes
NC7	8271_9	9bpDel	71												9bpDel									Yes
	8292	G-A	0															A						Yes
	8296	A-G	0	G																				Yes
ATP8	8392	G-A	5														A							Yes
	8414	C-T (Leu17Phe)	57		T		T		T	T		T	T									T		Yes
	8459	A-G(Asn32Asp)	3																G					Yes
ATP6	8584	G-A (Ala20Thr)	58															A		A				Yes
	8618	1		C																				Yes
	8701	A-G (Thr59Ala)	160		G		G		G	G		G	G		G	G		G		G		G		Yes
	8794	C-T(His90Tyr)	23																T					Yes
	8860	A-G (Thr112Ala)	334	G	G	G	G	G	G	G	G	G	G	G	G	G	G	G	G	G	G	G	G	Yes
	8928	T-C	0	C																				Yes
	8952	T-C	0												C									Yes
	9053	G-A (Ser176Asn)	25					A			A			A									A	Yes
	9090	T-C	9															C		C				Yes
	9180	A-G(Val218Val)	15	G																				Yes
CO3	9494	A-G	1					G						G										Yes
	9536	C-T	8				T		T	T		T	T											Yes
	9540	T-C	160		C		C		C	C		C	C			C		C		C		C		Yes
	9548	G-A	11					A						A										Yes
	9713	G-A	0															A						Yes
	9824	T-C	26													C								Yes
	9854	T-C	2																		C			Yes
ND3	10086	A-G	0																			G		Yes
	10188	A-G	0																				T	Yes
	10208	T-C	8															C						Yes
	10289	A-G	0	G																				Yes
	10310	G-A	56			A		A			A			A							A		A	Yes
	10320	G-A (Val88Ile)	8																		A			Yes
	10370	T-C	0														C							Yes
	10398	A-G (Thr114Ala)	193		G		G		G	G		G	G			G	G	G		G		G		Yes
	10400	C-T	162		T		T		T	T		T	T			T		T		T		T		Yes
ND4L	10499	A-G	1																		G			Yes
	10609	T-C (Met47Thr)	29					C			C			C									C	Yes
	10640	T-C	0												C									Yes
ND4	10873	T-C	164		C		C		C	C		C	C			C		C		C		C		Yes
	10908	0	C																				Yes	
	10909	T-C	0			C																		Yes
	10915	T-C	2			C																		Yes
	11065	A-G	7																		G			Yes
	11087	0					C						C										Yes	
	11204	T-C (Phe149Leu)	1												C									Yes
	11215	C-T	11				T		T	T		T	T											Yes
	11380	A-G	3					G						G										Yes
	11430	0																				G	No	
	11581	G-A	0	A																				Yes
	11719	G-A	327	A	A	G	A	A	A	A	A	A	A	A	A	A	A	A	A	A	A	A	A	Yes
	11776	T-C	1			C																		Yes
	11884	A-G	0													G								Yes
	11914	G-A	27														A							Yes
ND5	12361	A-G (Thr9Ala)	15														G							Yes
	12405	C-T	11													T								Yes
	12406	G-A (Val24Ile)	31					A			A			A									A	Yes
	12477	T-C	0		C																			Yes
	12612	A-G	6			G																		Yes
	12621	C-T	5																		T			Yes
	12630	G-A	7			A							A											Yes
	12684	G-A	0					A						A										Yes
	12705	C-T	209	T	T		T		T	T		T	T			T	T	T	T	T		T		Yes
	12771	G-A	6								A												A	Yes
	12819	A-G	0			G																		Yes
	12882	C-T	11					T			T			T									T	Yes
	13194	G-A	0														A							Yes
	13269	A-G	1												G									Yes
	13356	T-C	0		C																			Yes
	13416	A-G	0						T															Yes
	13620	T-C	0															C						Yes
	13759	G-A (Ala475Thr)	19					A			A			A										Yes
	13928	G-C (Ser531Thr)	47			C		C			C			C			C				C			Yes
	14016	A-G	1			G																		Yes
	14067	C-T	3																T					Yes
ND6	14178	T-C (Ile166Val)	5														C							Yes
	14220	A-G	0	G																				Yes
	14311	T-C	3	C																				Yes
	14431	T-C	0	C																				Yes
	14470	T-C	15				C		C	C		C	C											Yes
	14668	C-T	59		T		T		T	T		T	T									T		Yes
Cytb	14766	C-T (Thr7Ile)	339	T	T	T	T	T	T	T	T	T		T	T	T	T	T	T	T	T	T		Yes
	14783	T-C	165		C		C		C	C		C				C		C		C		C		Yes
	14971	T-C	4																		C			Yes
	15040	C-T	0																					Yes
	15043	G-A	91		A		A		A	A		A				A		A		A		A		Yes
	15110	G-A (Ala122Thr)	1													A								Yes
	15262	T-C	0																C					Yes
	15301	G-A	155		A		A		A	A		A				A		A				A		Yes
	15326	A-G (Thr194Ala)	333	G	G	G	G	G	G	G	G	G		G	G	G	G	G	G		G	G	G	Yes
	15475	A-G	0																	G				Yes
	15487	A-G	35															T		T				Yes
	15514	T-C(Tyr256Tyr)	0	C																				Yes
	15784	T-C	9															C		C				Yes
	15883	G-A	0														A							Yes
Thr	15900	T-C	1	C																				Yes
	15901	A-G	0		G																			Yes
	15908	T-C	1			C																		Yes
	15924	A-G	3				G	G	G	G	G	G	G	G	G									Yes
	15928	G-A	2													A	A	A						Yes
	15931	A-C	0																C					No
	15940	DelT	0																	Del				Yes
	15943	T-C	1																		C	C		Yes
	15949	G-A	0																				A	Yes

^a^According to MITOMAP (http://www.mitomap.org/MITOMAP). Database of reported mitochondrial DNA base substitution was last edited on January 1, 2019.

### Analysis of entire mtDNA sequences in probands

The past study examined the entire mtDNA sequences of 8 subjects consisted of five females and three males carrying the m.15927G > A mutation (Zhang et al. 2018). *MT-TT* m.15951A > G mutation may have a potential modifier role in increasing the penetrance and expressivity of the primary LHON-associated m.11778G > A mutation in a Chinese family (Li et al. 2006). In this study, we determined the complete mtDNA sequence analysis of additional four probands carrying m.15951A > G mutation. Furthermore, these probands exhibited distinct sets of mtDNA polymorphisms including 70 known variants. We further performed the haplogroup analysis of mtDNAs carrying the m.15951A > G mutation. As shown in Table 4, the mtDNAs from four Chinese families carrying the m.15951A > G mutation belong to Eastern Asian mtDNA haplogroup D. The frequency of mtDNA haplogroups D in 41 LHON families carrying the *MT-TT* mutation were 29.3%; while that of 376 Chinese controls was 21.5%. And then, we determined the complete mtDNA sequence analysis of additional 20 probands carrying putative LHON-associated variants, these probands exhibited distinct sets of mtDNA polymorphisms including 217 known variants and 4 unknown variants (Table 3). Thus, the frequency of haplogroup B in the Chinese pedigrees carrying the *MT-TT* mutations were significantly higher than that in 376 Chinese controls and other Asian populations. Meanwhile, that of haplogroup M8 was much lower than control individuals. This discrepancy between the different ethnic origins may be attributed to evolution.

**Table 4.** mtDNA haplogroup from 41 Han Chinese LHON probands carrying *MT-TT* variants and 376 control subjects.

Frequency of mtDNA haplogroup, %	Macrogroup M				Macrogroup N				
	D	G	M7	M8	A	B	N	H2	F
All subjects with *MT-TT* mutations, *n =* 41	29.3	4.9	2.4	4.9	2.4	31.7	2.4	4.9	17.1
Subjects with the m.15951A > G mutation, *n =* 4	100.0	0.0	0.0	0.0	0.0	0.0	0.0	0.0	0.0
Subjects with the m.15927G > A mutation, *n =* 8	0.0	25.0	0.0	0.0	0.0	62.5	0.0	0.0	12.5
Subjects with the putative *MT-TT*mutations, *n =* 20	28.6	0.0	4.8	4.8	0.0	28.6	4.8	0.0	28.6
Control, *n =* 376	21.5	4.3	6.9	10.6	6.6	18.6	8.8	0.8	16.0

## Discussion

The majority of patients with LHON (90%–95%) harbors one of three primary mtDNA point mutations, including m.3460G > A, m.11778G > A, and m.14484T > C, while these mutations are only responsible for 38.3% and 41.1% cases in two large cohorts of Chinese LHON subjects, respectively (Jia et al. 2006; Liang et al. 2014; Jiang et al. 2015; Ji et al. 2016). A number of LHON-associated mtDNA mutationts have been reported (Table 5), with some still awaiting full confirmation for pathogenicity, having been identified in only single families (Lott et al. 2013). *MT-TT* gene region is thought to be “mutational hotspot”, harboring other LHON-causing mutations, in addition to m.15927G > A and m.15951A > G (Li et al. 2006; Zhang et al. 2018). The coexistent of the m.15924A > G and m.3635G > A in some Chinese families indicate that m.15924A > G mutation may play a synergistic role in the phenotypic manifestation of LHON associated *MT-ND1* 3635G > A mutation (Zhang et al. 2014). The marked male bias and variability in the clinical phenotypes suggest nuclear modifier gene(s) or environmental factor(s) appear to play a role in the phenotypic expression in these 20 Chinese pedigrees (Yu-Wai-Man et al. 2011). Nuclear modifier genes were proposed to increase the susceptibility to LHON-associated mtDNA mutations (Chen et al. 2015). Three studies using microsatellite markers have confirmed significant linkage on the X-chromosome, with some of these candidate regions showing areas of overlap (Shankar et al. 2008; Ji et al. 2010). A genome-wide study of nine large m.11778G > A Thai pedigrees found two SNPs (rs3749446 and rs1402000), located within *PARL* (Presenilin-associated rhomboid-like) were associated with a statistically increased risk of phenotypic expression among LHON carriers (Phasukkijwatana et al. 2010). However, the association between these two *PARL* SNPs and visual loss was not replicated in an independent cohort of Chinese m.11778G > A LHON pedigrees (Zhang et al. 2010). In our previous study, we identified a mutation in *YARS2* as a nuclear modifier for the phenotypic manifestation of LHON-associated m.11778G > A mutation (Jiang et al. 2016).

**TABLE 5.** LHON-associated mtDNA mutations that have been reported in the Mitomap Website.

Top 21 primary LHON mutations			Other candidate LHON mutations		
Gene	Mutation	Amino acid change	Gene	Mutation	Amino acid change
*MT-ND4*	m.11778G > A	R340H	*MT-ND1*	m.3394T > C	Y30H
*MT-ND1*	m.3460G > A	A52T		m.3472T > C	F56L
	m.3866T > C	I187T		m.4025C > T	T240M
*MT-ND6*	m.14484T > C	M64V		m.4160T > C	L285P
*MT-ND1*	m.3376G > A	E24K	*MT-TM*	m.4435A > G	
	m.3635G > A	S110N	*MT-ND2*	m.4640C > A	I57M
	m.3697G > A	G131S		m.5244G > A	G259S
	m.3700G > A	A112T	*MT-ATP6*	m.9101T > C	I192T
	m.3733G > A	E143K	*MT-CO3*	m.9804G > A	A200T
	m.4171C > A	L289M	*MT-ND3*	m.10237T > C	I60T
*MT-ND3*	m.10197G > A	A47T	*MT-ND4*	m.11253T > C	I165T
*MT-ND4L*	m.10663T > C	V65A		m.11696G > A	V312I
*MT-ND5*	m.12338T > C	M1T	*MT-ND5*	m.12811T > C	Y159H
	m.13051G > A	G239S		m.12848C > T	A171V
	m.13094T > C	V253A		m.13637A > G	Q434R
*MT-ND6*	m.14459G > A	A72V		m.13730G > A	G465E
	m.14482C > A	M64I	*MT-ND6*	m.14279G > A	S132L
	m.14482C > G	M64I		m.14325T > C	N117D
	m.14495A > G	L60S		m.14498T > C	Y59C
	m.14502T > C	I58V		m.14596A > T	I26M
* *	m.14568C > T	G36S	*MT-TE*	m.14693A > G	
			*MT-Cytb*	m.14831G > A	A29T
			*MT-TT*	m.15927G > A	
			* *	m.15951A > G	

In the present study, using the Sanger sequence of *MT-TT* gene, we identified 15 known variants in *MT-TT* gene were identified in a cohort of 352 Han Chinese subjects with LHON. These variants could have potential structural alterations and functional significance of *MT-TT*. In particular, these variants could affect the processing of the tRNAs from the primary transcripts, stability of the folded secondary structure, the charging of the tRNA, or the codon-anticodon interaction in the process of translation. Seven variants at tRNA stems, abolishing the Watson-Crick (WC) base pairs of mitochondrial tRNAs, likely lead to the tRNA aminoacylation, editing, and modification, which might result in low efficiency and accuracy of mitochondrial protein synthesis (Wang et al. 2018). The m.15908T > C mutation affected a highly conserved thymine at position 23 at the DHU-stem of *MT-TT*, destabilizing the conservative base pairing (12A-23T). That may alter the secondary structure and function of *MT-TT*, Two mutations at the acceptor stem 15951A > G and 15949G > A may alter the secondary structure and function of tRNAs, as in the case of the *MT-TS* 7511T > C (A4) (Li et al. 2006) and *MT-TH* 12201T > C (U68) (Gong et al. 2014) mutations. Moreover, two variants (m.15924A > G and m.15928G > A) at the anticodon stem may affect the function of tRNAs, as in the case of the *MT-TT* 15927G > A mutation (Jia et al. 2018; Zhang et al. 2018). Finally, m.15943T > C at the T-stem may also affect the structure and function of tRNAs. However, the functional significances of these putative LHON-associated tRNA variants should be further investigated.

The failures in tRNA metabolisms caused by these putative LHON-associated variants would lead to the impairment of mitochondrial protein synthesis and deficient respirations, as in the case of other mitochondrial tRNA mutations (Li et al. 2006; Jiang et al. 2016; Xue et al. 2016; Jia et al. 2018; Zhang et al. 2018). The *MT-TE* m.14693A > G variant may act as modifiers influencing the phenotypic manifestation of LHON-associated m.3460G > A mutation (Tong et al. 2007). Furthermore, our previous investigation showed that *MT-TM* m.4435A > G and *MT-TT* m.15951A > G mutations modulate the phenotypic expression of the LHON-associated m.11778G > A mutation in Chinese families (Li et al. 2006; Qu et al. 2006). However, the tissue specificity of these tRNA variants is likely attributed to tissue-specific tRNA metabolism or the involvement of nuclear modifier genes (Dittmar et al. 2006; Chen et al. 2016). The homoplasmic nature of these mitochondrial tRNA variants hints to mild nature of mutations. These suggest that the tRNA variants may be insufficient to produce a clinical phenotype by itself but the inherited risk factor(s) is necessary for the development of LHON. Nuclear modifier genes, environmental and epigenetic factors, as well as personal lifestyles such as smoking and drinking may also contribute to the development of LHON in these subjects carrying the mtDNA variants (Carelli et al. 2016; Jiang et al. 2016).

Here, mtDNAs in 41 LHON families carrying the *MT-TT* variants were widely dispersed among 9 Eastern Asian subhaplogroups. Indeed, the occurrences of mtDNA haplogroups D in families carrying the m.15951A > G mutation were higher than those in controls. Moreover, the frequencies of mtDNAs in haplogroups G and B in eight Chinese families carrying the m.15927G > A mutation, while 20 pedigrees carrying one of nine putative variants were similar to those in controls. Thus, the frequencies of haplogroups G, B, and F in the LHON probands carrying the *MT-TT* mutations were significantly higher than those in 478 Chinese controls and other Asian populations (Tanaka et al. 2004; Kong et al. 2006). mtDNA haplogroups M7b1’2 and M8a affect clinical expression of LHON in Chinese families with the m.11778G > A Mutation (Ji et al. 2008). This discrepancy implicates a role of mtDNA haplotypes in the phenotypic manifestation of LHON-associated mtDNA mutations (Liang et al. 2014; Jiang et al. 2015; Ji et al. 2016).

In summary, this is the first study to investigate the frequency and spectrum of mutations in *MT-TT* gene in Chinese subjects with LHON. The two known LHON-associated *MT-TT* mutations, m.15927G > A and m.15951A > G, in Chinese cohort accounted for 3.41% cases of 352 Chinese subjects with LHON. Furthermore, the nine putative LHON-associated mtDNA variants were the rare mutations, accounting for 5.66% cases in this Chinese cohort. A total of 41 subjects carrying one of the *MT-TT* mutations accounted for 9.07% cases of 352 Chinese subjects with LHON. These data further support that the *MT-TT* gene is a hotspot for mutations associated with LHON. Thus, our findings may provide valuable information for the further understanding of pathophysiology and management of LHON.

## Data Availability

The analyzed data and materials generated during the study are available from the corresponding author on reasonable request.
